# Impact of Gentamicin‐Impregnated Collagen Sponge on the Incidence of Access Related Infections After Full‐Support Microaxial Flow Pump Therapy

**DOI:** 10.1111/aor.70141

**Published:** 2026-04-26

**Authors:** Gaik Nersesian, Maximilian Rauch, Daniel Lewin, Leonard Pitts, Julius Kaemmel, Anna Stegmann, Julia Stein, Yuriy Hrytsyna, Felix Schoenrath, Volkmar Falk, Evgenij Potapov, Pia Lanmueller

**Affiliations:** ^1^ Department of Cardiothoracic and Vascular Surgery Deutsches Herzzentrum der Charité (DHZC) Berlin Germany; ^2^ DZHK (German Centre for Cardiovascular Research), partner Site Berlin Berlin Germany; ^3^ Berlin Institute of Health at Charité ‐ Universitätsmedizin Berlin Berlin Germany; ^4^ Department of Health Sciences and Technology Institute of Translational Medicine, Translational Cardiovascular Technologies, Swiss Federal Institute of Technology (ETH) Zurich Zurich Switzerland

**Keywords:** brachial plexus lesion, cardiogenic shock, Impella, microaxial flow pump, prosthetic graft infection

## Abstract

**Background:**

Surgically implanted microaxial flow pumps (mAFP) are increasingly used for cardiogenic shock treatment. Standard mAFP explantation is performed bedside with a shortening of the vascular prosthesis, which may represent a potential source of infection in future. We analyzed the impact of gentamicin‐impregnated collagen sponge (GICS) application in the wound during mAFP explantation on the incidence of prosthetic graft infections.

**Methods:**

Between 01/2020 and 07/2024, 235 patients underwent bedside full‐support mAFP explantation at our institution. Since 11/2022, GICS has been routinely applied in 115 patients, while 120 previously were treated without it.

**Results:**

In the GICS‐group, after a median follow‐up of 221 [24; 351] days, 17 (14.8%) patients developed graft infection, resulting in 0.23 events per patient year (EPPY); surgery was necessary in 16 patients (13.9%). In the control group, after a median follow‐up of 399 [113; 654] days, infection occurred in 14 (11.7%) cases, 0.10 EPPY; surgical removal of the graft was necessary in 12 (10%). The GICS‐groups presented an increased risk for graft infections: sHR 2.49 [1.11; 5.59], *p* = 0.027. After propensity score matching for relevant demographic parameters, there was no significant difference in the risk reduction of total graft infections sHR 1.92 [0.79; 4,72], *p* = 0.15.

**Conclusions:**

Local application of GICS did not reduce the risk of access site infections in patients undergoing bedside explantation of mAFP from the graft surgically anastomosed to an axillary artery.

## Graphical abstract

Local application of gentamicin for wound closure after full‐support microaxial flow pump therapy.

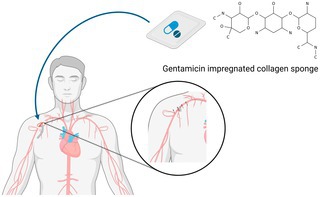

**Abbreviations**
dLVAD: durable left ventricular assist device
EPPY: events per patient year
GICS: gentamicin‐impregnated collagen sponge
HR: hazard ratio
IQR: interquartile range
mAFP: microaxial flow pump
PGI: prosthetic graft infection
SD: standard deviation
SHR: subdistributional hazard ratio
SMD: standardized mean difference
tMCS: temporary mechanical circulatory support
VA‐ECLS: veno‐arterial extracorporeal life support

## 1 Introduction

Microaxial flow pumps (mAFP) have been increasingly used for temporary mechanical circulatory support (tMCS) in cardiogenic shock patients [1]. The standard implantation technique of full‐support mAFPs includes an anastomosis of a 10‐mm or 8‐mm vascular graft prosthesis to the axillary artery, which is used for pump insertion and fixation [1]. In cases of myocardial recovery, or bridging to a durable left ventricular assist device (dLVAD) or heart transplantation, the mAFP is removed. The standard explantation technique involves shortening, ligation, and partial removal of the graft prosthesis. This approach avoids complete re‐opening of the wound and potential damage to surrounding structures, but leaves a small portion of the graft remaining in the patient's body attached to the artery [2].

The remaining portion of the vascular graft in the wound carries a potential risk for access site infections occurring in approximately 10% of surgically implanted axillary‐cannulated mAFP devices [3]. At the same time, reopening of the wound and complete explantation of the vascular graft prosthesis requires a meticulous surgical exposure of the axillary artery and carries a potential risk of damage to the brachial plexus, axillary artery, or vein. Therefore, complete removal of the vascular graft prosthesis is not performed routinely in every case of surgical mAFP explantation and is reserved for patients with early complications (e.g., infections, thromboembolic complications, dissections) and for bridge‐to‐transplant candidates [3].

Gentamicin‐impregnated collagen sponges (GICS) have demonstrated promising results in reducing the risk of sternal wound infections in patients undergoing cardiac surgery [4, 5]. The application of a local antibiotic agent has been considered a promising concept, as it allows the delivery of high doses of medication directly to the infected area while avoiding elevated blood concentrations and associated systemic toxicity [6]. In this context, GICS represents a versatile agent, since it covers a wide patter of gram‐negative pathogens as well as important gram‐positive bacteria, including *Staphylococcus aureus* and coagulase‐negative *Staphylococci* [4].

In presented study, we evaluate the impact of GICS application on the incidence of access related infections in patients who underwent explantation of surgically‐placed axillary mAFP with a residual portion of a vascular graft left in the wound.

## 2 Patients and Methods

**FIGURE 1:**
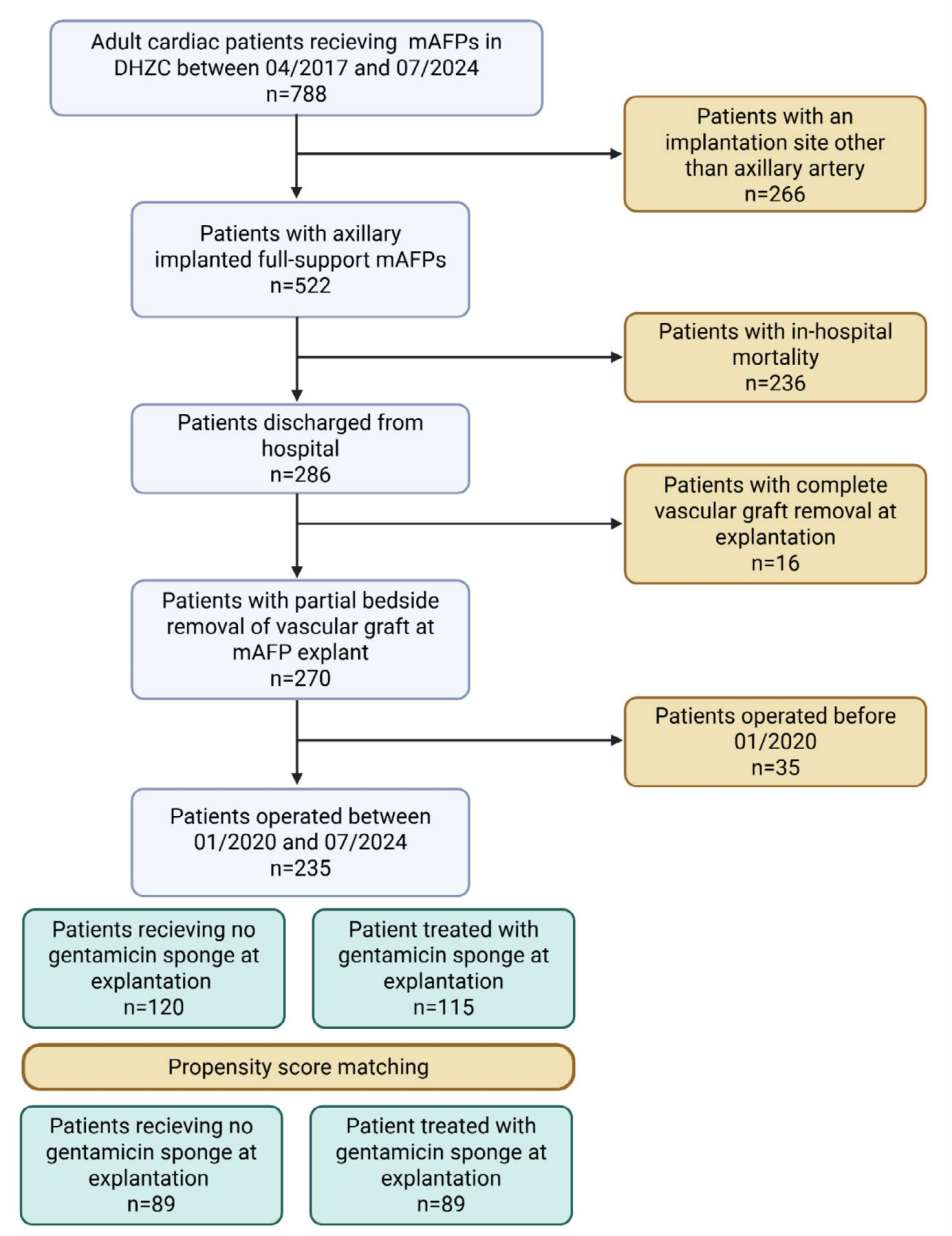
Flow‐chart of study population. mAFP, microaxial flow pump. [Color figure can be viewed at wileyonlinelibrary.com]

The present study is a retrospective, observational, propensity score‐matched analysis including full‐support mAFP patients from the Deutsches Herzzentrum der Charité, aimed at evaluating the impact of local application of GICS on the incidence of prosthetic graft infections (PGI) after bedside mAFP explantation (Figure 1).

Patients who underwent total surgical prosthetic graft removal during mAFP explant were excluded from the analysis. To reduce the impact of differing time periods between cohorts and make them more homogeneous, patients who underwent mAFP implantation before 01/01/2020 were excluded prior to propensity‐score matching (Figure 1).

### 2.1 Endpoints

Primary endpoint of the study was the incidence of PGI. Secondary endpoints included PGIs requiring surgical revision, incidence of brachial injury after surgical revision of PGI, and overall survival.

### 2.2 Ethical Votum

The study was approved by our institutional ethic committee (EA1/215/25).

### 2.3 Surgical Technique

For surgical mAFP implantation, the axillary artery was exposed via a 5 cm longitudinal incision over the infraclavicular fossa. 5000–7000 IE of unfractioned Heparin were administrated aiming for an activated clotting time (ACT) of 250 s. The artery was partially clamped, and a 10 mm Hemashield graft (MA‐QUET Ltd., Rastatt, Germany) was anastomosed in end‐to‐side fashion using a 5‐0 running Prolene suture. The graft was carried out of the wound through a separate skin incision approximately 5 cm below the initial access site. The pump was advanced through the graft and positioned in the left ventricle using a combination of fluoroscopic and echocardiographic guidance. A 12‐Fr drainage tube was placed at the primary access site, and the wound was closed in layers [7]. A purse suture was placed at the graft exit site and tightened around the graft in order to avoid wound exposure. Sterile wound dressing was applied at the wound and external portion of the graft.

At our institution, bedside mAFP explantation under local anesthesia is performed as the standard approach. The mAFP is stopped and removed from the graft prosthesis. The graft is shortened, meticulously ligated, and pushed beneath the pectoral muscle. In the GICS group, the impregnated collagen sponge is inserted into the wound channel prior to the skin closure.

In cases involving bridge‐to‐transplant patients or early prosthetic graft infection, the primary wound in the deltopectoral sulcus is re‐opened, and the axillary artery with graft prosthesis is exposed. The prosthetic material is completely removed, and the axillary artery is reconstructed using a bovine pericardial patch.

### 2.4 Prosthetic Graft Infection

**FIGURE 2:**
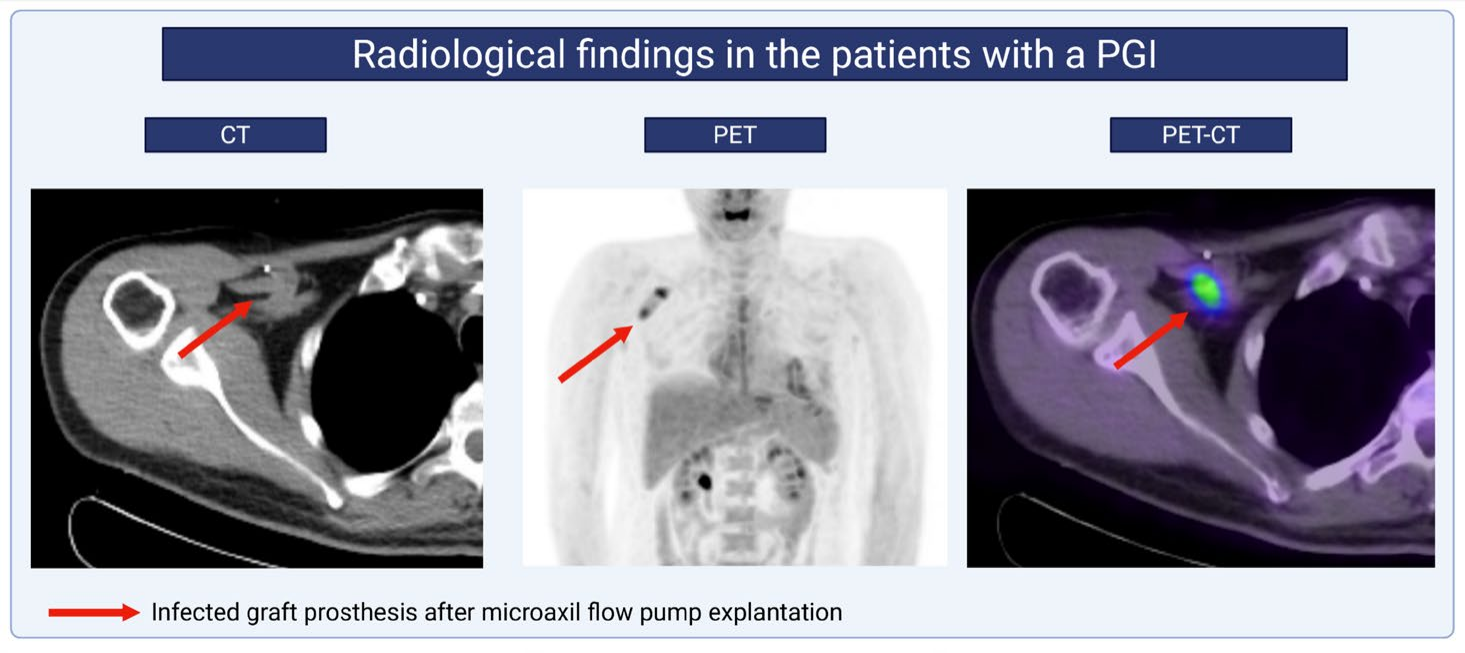
Radiological findings in the patient with a PGI. [Color figure can be viewed at wileyonlinelibrary.com]

The diagnosis of PGIs was based on clinical findings including systemic signs such as fever, bacteremia or leucocytosis, and/or local signs including characteristics of inflammation (rubor, calor, tumor, dolor, functio laesa), wound dehiscence, or purulence [3]. Laboratory and microbiological diagnostics were performed. Computed tomography and in some cases positron‐emission tomography/computed tomography (PET/CT‐Scan) were performed for confirmation of diagnosis and surgical planning (Figure 2).

### 2.5 Statistical Analysis

**FIGURE 3:**
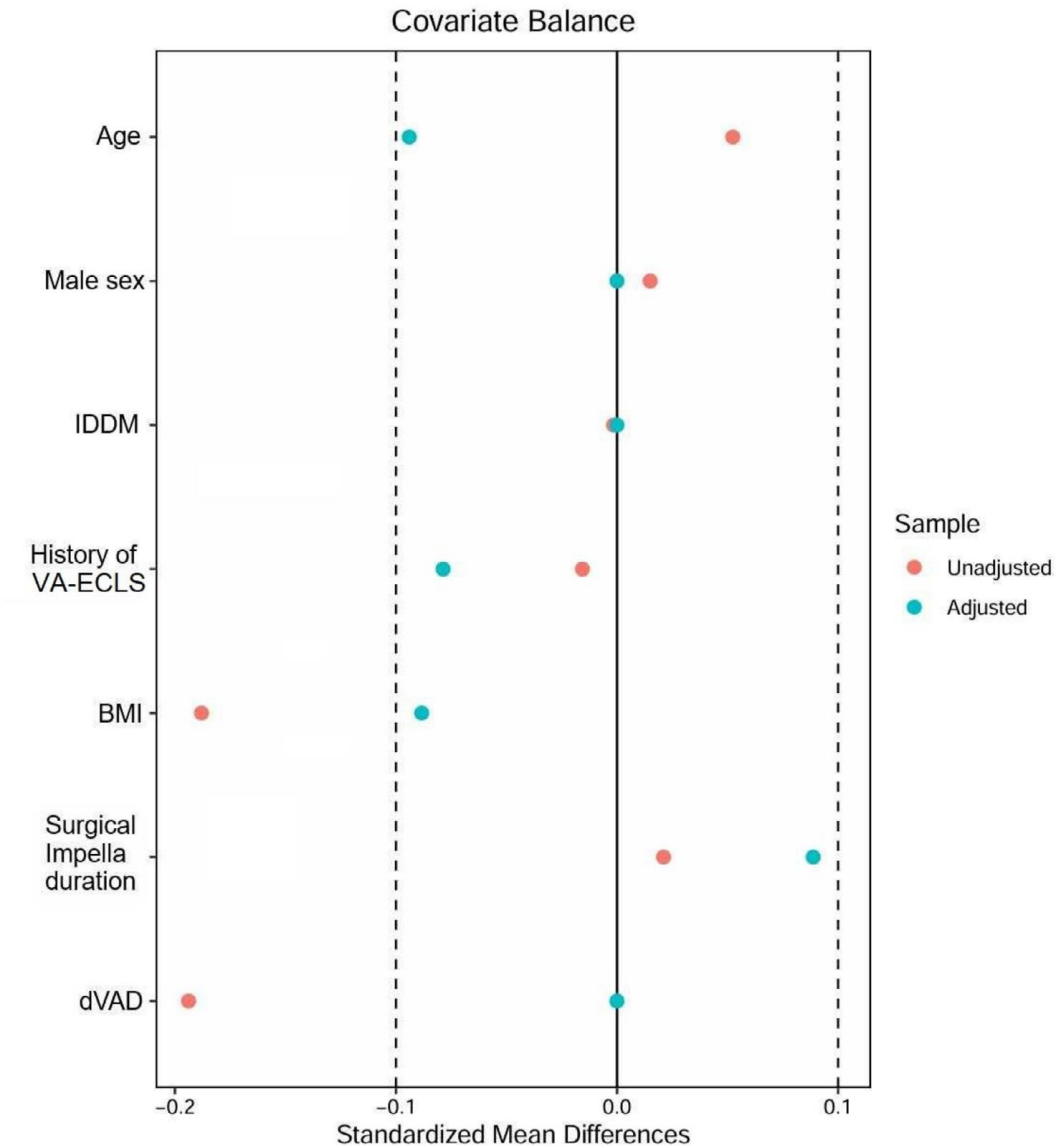
Balance plot for matching parameters. BMI, body mass index; dVAD, durable ventricular assist device; IDDM, insulin‐dependent diabetes mellitus; VA‐ECLS, veno‐arterial extracorporeal life support. [Color figure can be viewed at wileyonlinelibrary.com]

For baseline characteristics, continuous variables are summarized as mean and standard deviation (SD), or as median and interquartile range (IQR) (25th quantile–75th quantile) in the case of skewed data. For categorical variables, numbers and percentages are reported (Table 1). To account for imbalances in the gentamicin and control patient groups, a propensity score was calculated by logistic regression. Parameters used for propensity score matching included age, sex, body mass index, insulin dependent diabetes mellitus, extracorporeal life support, duration of mAFP support, and durable LVAD implantation. The propensity scores were estimated using a General Linear Model with a logit link function. Groups were matched using the nearest‐neighbor algorithm without replacement and a caliper width of 0.15. The balance was verified by means of the standardized mean difference (Table 2) and is presented graphically in a balance plot (Figure 3).

**TABLE 1:** Patient's preoperative demographics.

Parameter	Total	Control cohort	Gentamicin cohort	*p*	SMD	Missing (%)
Number of patients	235	120	115			
Age (mean (SD))	57.5 (13)	57.3 (13.2)	57.8 (12.9)	0.75	0.042	0
Male sex (%)	195 (83.0)	99 (82.5)	96 (83.5)	0.98	0.026	0
Weight (mean (SD))	86.2 (22.2)	87.7 (20.1)	84.6 (24.2)	0.29	0.139	2.1
Height (mean (SD))	176.4 (8.4)	176.1 (7.5)	176.7 (9.4)	0.58	0.074	3.0
BMI (mean (SD))	27.65 (6.48)	28.27 (6.37)	26.97 (6.56)	0.13	0.201	3.0
CIED (%)	55 (23.8)	28 (23.7)	27 (23.9)	1.0	0.004	1.7
History of Afib. (%)	62 (27.0)	35 (29.9)	27 (23.9)	0.38	0.14	2.1
DM (%)	79 (34.5)	44 (37.9)	35 (31.0)	0.33	0.15	2.6
IDDM (%)	23 (10.0)	12 (10.3)	11 (9.7)	1.0	0.02	2.6
CKD (%)	48 (21.1)	30 (26.1)	18 (16.1)	0.09	0.25	3.4
COPD (%)	24 (10.8)	11 (9.8)	13 (11.3)	0.81	0.06	5.1
Pulmonary hypertension (%)	15 (6.8)	11 (10.0)	4 (3.6)	0.25	0.19	0
History of stroke (%)	8 (3.4)	2 (1.7)	6 (5.2)	0.25	0.20	0
Arterial hypertension (%)	129 (57.6)	73 (64.0)	56 (50.9)	0.06	0.27	4.7
History of AMI (%)	67 (39.9)	41 (45.1)	26 (33.8)	0.18	0.23	28.5
CPR (%)	17 (10.1)	11 (12.1)	6 (7.8)	0.51	0.14	28.5
History of va‐ECMO (%)	100 (42.6)	52 (43.3)	48 (41.7)	0.91	0.032	0
Invasive ventilation (%)	129 (77.7)	65 (72.2)	64 (84.2)	0.10	0.30	29.4
Preoperative RRT (%)	10 (6.1)	6 (6.7)	4 (5.3)	0.98	0.06	29.8
Nicotine consumption (%)	123 (55.2)	65 (59.1)	58 (51.3)	0.30	0.16	5.1
Alcohol consumption (%)	52 (23.9)	30 (28.6)	22 (9.5)	0.16	0.21	7.2
Preoperative inotropes (%)	226 (96.2)	114 (95.0)	112 (97.4)	0.54	0.13	0.0
Heart failure etiology						20.0
ICMP (%)	68 (36.2)	35 (34.3)	33 (38.4)	0.67	0.08	20.0
AMI (%)	31 (16.5)	17 (16.7)	14 (16.3)	1.0	0.01	20.0
DCMP (%)	63 (33.5)	36 (35.3)	27 (31.4)	0.68	0.08	20.0
Postcardiotomy (%)	32 (18.8)	21 (22.8)	11 (14.1)	0.21	0.23	27.7
Other (%)	10 (5.3)	4 (3.9)	6 (7.0)	0.55	0.14	20.0
Failure to wean from CPB (%)	7 (23.3)	5 (25.0)	2 (20.0)	1.0	0.12	0

Parameter	Total	Control cohort	GICS cohort	*p*
ICU duration (days, median, IQR)	23 [13, 36]	26 [13, 39]	19 [11, 33]	0.13
Orotracheal extubation (%)	164 (72.6)	76 (66.1)	88 (79.3)	0.04
Tracheostomy (%)	46 (20.4)	28 (24.3)	18 (16.2)	0.18
ICU discharge (%)^a^	136 (57.9)	57 (47.5)	79 (68.7)	0.001
In‐hospital stay (days, median, IQR)^a^	29 [19, 46]	32 [22, 48]	27 [18, 44]	0.33
Discharge home (%)^a^	106 (45.1)	79 (65.8)	27 (23.5)	< 0.001
Graft infection (%)	31 (13.2)	14 (11.7)	17 (14.8)	0.55
Graft infection requiring surgery (%)	28 (11.2)	12 (10)	16 (13.9)	0.35

Abbreviations: Afib, arterial fibrillation; AMI, acute myocardial infarction; BMI, body mass index; CIED, cardiac implantable electronic device; CKD, chronic kidney disease; COPD, chronic pulmonary disease; CPB, cardiopulmonary bypass; CPR, cardiopulmonary resuscitation; DCMP, dilative cardiomyopathy; DM, diabetes mellitus; GICS, gentamicin impregnated collagen sponge; ICMP, ischemic cardiomyopathy; IDDM, insulin dependent diabetes mellitus; RRT, renal replacement therapy; SMD, standard mean deviation; va‐ECMO, veno‐arterial extracorporeal membrane oxygenation.

^a^ Refers to index admission time at our center or discharge from it; patients transferred to other hospitals are not included.

**TABLE 2:** Parameters after matching and postoperative outcomes.

Parameter	Total	Control cohort	GICS cohort	SMD
Number of patients	178	89	89	
Age (mean (SD))	58.18 (12.76)	58.79 (12.32)	57.57 (13.23)	0.095
Male sex (%)	150 (84.3)	75 (84.3)	75 (84.3)	< 0.001
IDDM (%)	18 (10.1)	9 (10.1)	9 (10.1)	< 0.001
va‐ECMO (%)	71 (39.9)	39 (43.8)	32 (36.0)	0.161
BMI (mean (SD))	27.07 (5.87)	27.36 (5.42)	26.77 (6.31)	0.099
dVAD (%)	66 (37.1)	33 (37.1)	33 (37.1)	< 0.001
Impella duration (days, median, IQR)	9 [5, 16]	9 [5, 16]	10 [6, 16]	0.089

				*p*
ICU duration (days, median, IQR)	22 [12, 37]	19 [12, 33]	24 [13, 39]	0.31
Orotracheal extubation (%)	124 (69.7)	56 (62.9)	68 (76.4)	0.08
Tracheostomy (%)	36 (20.2)	21 (23.6)	15 (16.9)	0.31
ICU discharge (%)^a^	107 (60.1)	42 (47.2)	65 (73)	0.001
In‐hospital stay (days, median, IQR)^a^	29 [19, 47]	27 [18, 45]	32 [20, 47]	0.51
Discharge home (%)^a^	83 (46.6)	59 (66.3)	24 (27)	< 0.001
Graft infection (%)	24 (13.5)	11 (12.4)	13 (14.6)	0.82
Graft infection requiring surgery (%)	21 (11.8)	8 (9)	13 (14.6)	0.63

Abbreviations: BMI, body mass index; dVAD, durable ventricular assist device; GICS, gentamicin impregnated collagen sponge; ICU, intensive care unit; IDDM, insulin dependent diabetes mellitus; IQR, interquartile range; SMD, standard mean deviation; va‐ECMO, veno‐arterial extracorporeal membrane oxygenation.

^a^ Refers to index admission time at our center or discharge from it; patients transferred to other hospitals are not included.

Competing risk analyses were used to evaluate the incidence of PGIs with death as competing outcomes. In the case of recurrent adverse events, the first event in a patient was analyzed. Subdistributional hazard ratios (sHRs) were calculated using clustered Fine–Gray models.

Survival was graphically displayed in a Kaplan–Meier plot and for estimating the influence of patient group on survival, the hazard ratio (HR) is given.

For sensitivity analysis, we performed a multivariable competing risk model.

R version 4.0.3 [R development Core team (2020). R: A Language and Environment for Statistical Computing] was used for statistical analysis.

## 3 Results

The demographic parameters in the GICS‐group (*n* = 115 patients) were comparable to control group (*n* = 120 patients): the mean age was 57.8 ± 12.9 years versus 57.3 ± 13.2 years, 96 (83.5%) versus 99 (82.5%) were male, 48 (41.7%) versus 52 (43.3%) had a history of VA‐ECLS treatment at index event (Table 1). Median support duration was 9 [6;15] versus 9 [5.8;16] days, 36 (31%) versus 60 (50%) underwent durable LVAD implantation; one patient was directly bridged to a heart transplant.

In the control group after a median follow‐up of 399 [113; 654] days, infection occurred in 14 (11.7%) cases, 0.10 EPPY, surgical removal of the graft was necessary in 12 (12%). In the GICS‐group after a median follow‐up of 221 [24; 351] days, 17 (14.8%) patients developed graft infection, resulting in 0.23 events per patient year (EPPY); surgery was necessary in 16 patients (13.9%).

**FIGURE 4:**
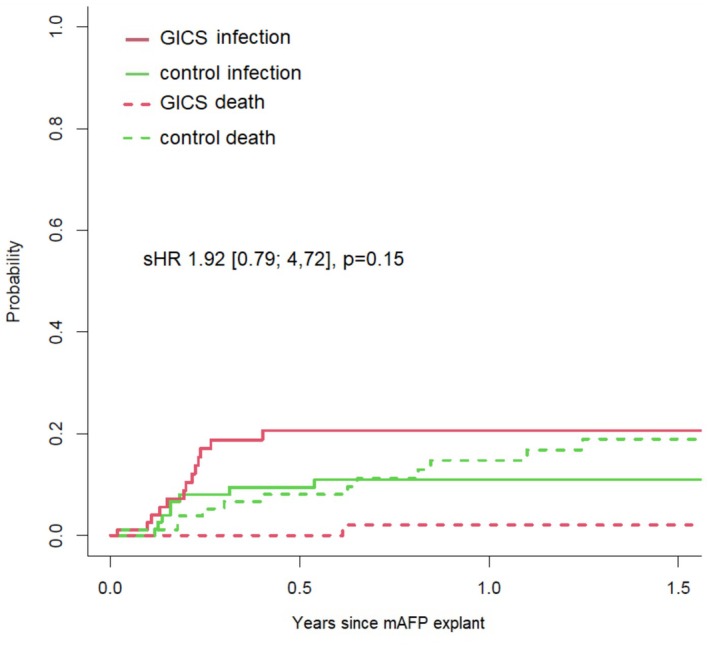
Cumulative incidence function for prosthetic graft infections between the groups. GICS, gentamicin‐impregnated collagen sponge; mAFP, microaxial flow pump; sHR, subdistributional hazard ratio. [Color figure can be viewed at wileyonlinelibrary.com]

The GICS‐groups presented an increased risk for graft infections: sHR 2.49 [1.11; 5.59], *p* = 0.027. After propensity score matching for relevant demographic parameters, there was no significant difference in the risk reduction of total graft infections sHR 1.92 [0.79; 4,72], *p* = 0.15, and those requiring surgery sHR 1.27 [0.47; 3,55], *p* = 0.63, between the groups (Figure 4).

There was no difference in the median time to infection 80 [58; 407] versus 76 [50; 87] days *p* = 0.26 and EPPY after matching 0.11 95% CI [0.06; 0.20] for control versus 0.23 95% CI [0.13; 0.37] for GICS‐groups, respectively.

**FIGURE 5:**
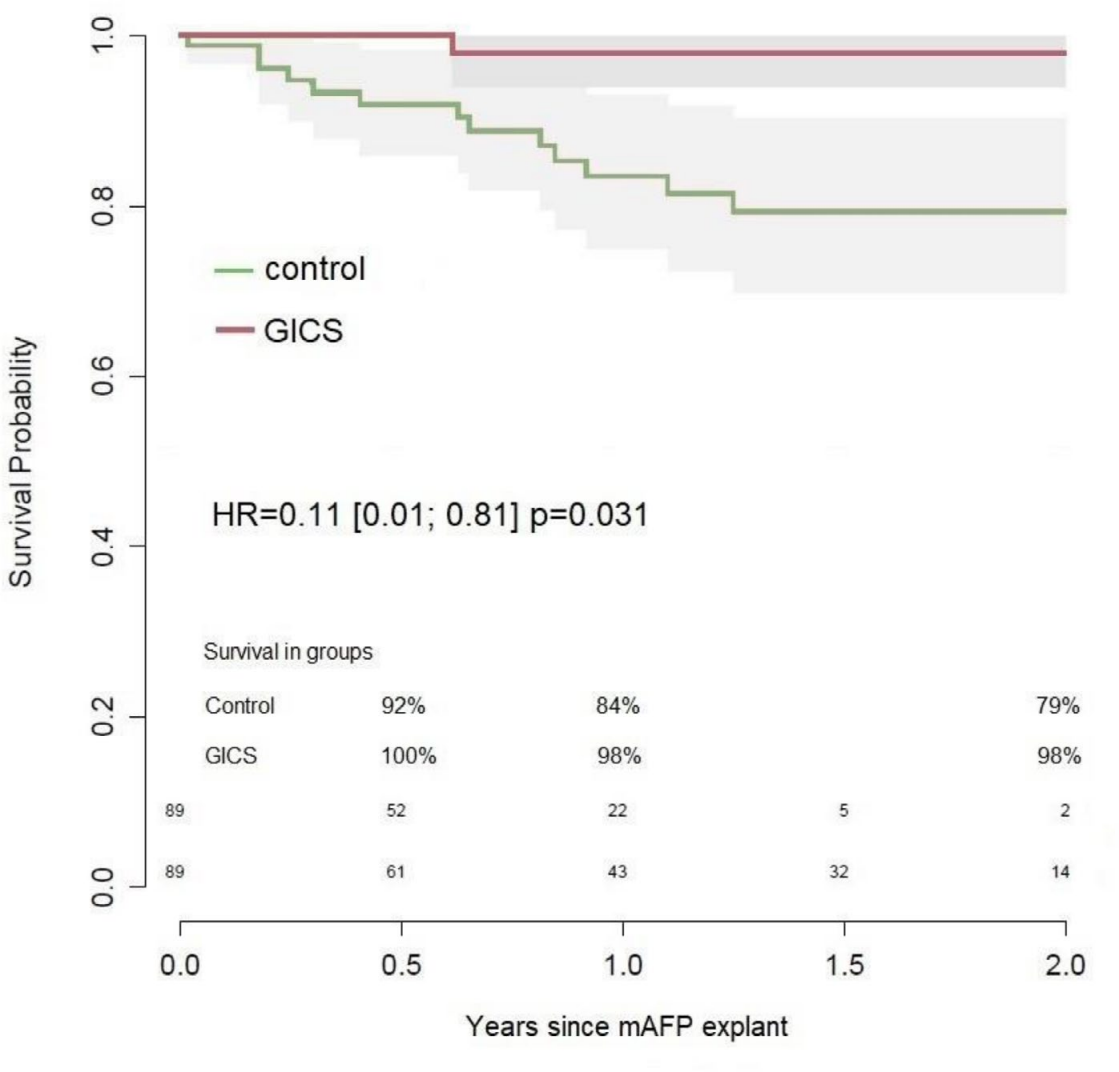
Kaplan–Meier estimates for survival probability between the groups. GICS, gentamicin‐impregnated collagen sponge; HR, subdistributional hazard ratio; mAFP, microaxial flow pump. [Color figure can be viewed at wileyonlinelibrary.com]

Patients in the GICS‐group presented a significantly higher survival probability on follow‐up: HR = 0.11 [0.01; 0.81] *p* = 0.031 (Figure 5).

Pathogen distribution and antibiotic treatment in matched and unmatched patients with PGI are presented in Table 3. Causative pathogens could be identified only in 13/31 (42%) of unmatched and 12/24 (50%) of matched patients and were predominantly represented by gram‐positive skin flora (Table 3). After complete surgical removal of graft remnants in PGI patients, brachial plexus injury (defined as sensory and/or motor deficits) were observed in 10/28 (36%) of the unmatched and 8/21 (38%) of the matched patients.

**TABLE 3:** Cases of prosthetic graft infections in matched and unmatched patients.

Infection case Nr.	Time to infection from explant (days)	Abscess formation	Pus/wound exudation	GICS	Surgical graft removal	Brachial plexus injury after revision	Pathogens	Antibiotic treatment	Duration of antibiotics (days)
**1**	**131**	**1**	**0**	**0**	**1**	**0**	***Staph. epidermidis* (MRSE)**	**Flucloxacillin, Fosfomycin**	**7**
2	104	1	0	0	1	0	Not identified	ND	14
**3**	**43**	**1**	**1**	**0**	**1**	**0**	**Staph. epidermidis**	**Linezolid**	**14**
**4**	**86**	**1**	**1**	**0**	**1**	**0**	**Not identified**	**Piperacillin/Tazobactam, Fosfomycin, Cotrimocxazol**	**14**
**5**	**21**	**0**	**0**	**0**	**1**	**0**	**Not identified**	**ND**	**ND**
**6**	**19**	**0**	**0**	**0**	**0**	**0**	**Not identified**	**ND**	**5**
**7**	**19**	**1**	**1**	**0**	**1**	**1**	**Not identified**	**ND**	**6**
**8**	**12**	**0**	**0**	**0**	**1**	**0**	**Staph. epidermidis**	**Ceftriaxon, Linezolid**	**14**
**9**	**78**	**1**	**0**	**0**	**1**	**0**	**Not identified**	**Piperacillin/Tazobactam, Vancomycin**	**10**
**10**	**47**	**1**	**0**	**0**	**0**	**1**	***Klebsiella aerogenes***	**Piperacillin/Tazobactam, Moxifloxacin**	**20**
11	115	1	0	1	1	1	Not identified	Meropenem, Vancomycin	2
12	28	0	0	1	1	0	Not identified	Linezolid, Cefuroxim	27
13	23	1	1	1	1	0	*Pseudomonas aeruginose*, *Klebsiella pneumoniae*	Meropenem	24
**14**	**20**	**1**	**0**	**1**	**1**	**0**	***Staph. aureus***	**Linezolid, Tazobactam, Cefazolin**	**21**
**15**	**68**	**0**	**1**	**1**	**1**	**0**	**Not identified**	**ND**	**3**
16	44	1	1	1	1	0	Not identified	ND	13
**17**	**10**	**1**	**1**	**1**	**1**	**0**	**Not identified**	**ND**	**48**
**18**	**ND**	**1**	**0**	**1**	**1**	**1**	**Not identified**	**ND**	
**19**	**57**	**0**	**1**	**1**	**1**	**1**	***Staph. capitis*, *Staph. haemolyticus*, *Corynebacterium tuberculostearicum* , *Staph. epidermidis***	**Vancomycin**	**10**
**20**	**45**	**1**	**1**	**1**	**1**	**1**	***Staph. epidermidis***	**Vancomycin**	**23**
**21**	**32**	**1**	**1**	**1**	**1**	**0**	**Not identified**	**Cefotaxim, Cefuroxim**	
**22**	**9**	**0**	**0**	**1**	**0**	**0**	***Cutibacterium acnes***	**Clyndamicin**	**30**
**23**	**ND**	**0**	**0**	**1**	**1**	**1**	**Not identified**	**ND**	**ND**
**24**	**62**	**0**	**0**	**1**	**1**	**1**	***Streptococcus pyogenes***	**ND**	**21**
**25**	**ND**	**1**	**1**	**1**	**1**	**1**	***Staph. epidermidis***	**Clyndamicin**	**ND**
26	3	0	0	0	1	0	Not identified	ND	25
**27**	**186**	**0**	**0**	**0**	**1**	**0**	**Not identified**	**Ampicillin/Sulbactam**	**24**
**28**	**49**	**1**	**0**	**0**	**1**	**0**	**Not identified**	**ND**	**ND**
29	150	1	0	0	1	1	Not identified	Piperacillin/Tazobactam, Meropenem	ND
**30**	**60**	**0**	**0**	**1**	**1**	**1**	***Pantoea agglomerans***	**Piperacillin/Tazobactam, Gentamicin**	**15**
**31**	**5**	**0**	**0**	**1**	**1**	**0**	***Staph. epidermidis*, *Staph. haemolyticus***	**Daptomycin**	**16**

*Note:* Bold marked are cases in matched cohorts.

Abbreviations: Nr., number; ND, no data; Staph., *Staphylococcus* species.

## 4 Comment

The presented results have demonstrated that the prophylactic application of GICS does not reduce the risk of PGIs in patients undergoing bedside explantation of surgically implanted full‐support mAFPs.

The application of local antibiotic agents in surgical patients remains a topic of high debate among representatives of different surgical disciplines [8]. In septic orthopedic surgery local application of antibiotic‐coated agents (especially gentamicin) is a widely established method for the treatment of osteomyelitis; however, the scientific evidence supporting this approach remains inconclusive [8].

In cardiac surgery, application of GICS for the prevention of sternal wound infections in patients undergoing median sternotomy has been highly debated [5]. Results from large randomized controlled trials have not demonstrated a clear benefit of local GICS application regarding the risk of sternal wound infections [9, 10]. However, the findings of a large meta‐analysis suggest some benefit in favor of GICS in patients with high‐risk profiles for wound infections (e.g., obesity, diabetes) [10]. One important critical point of previously performed studies might be the saline soaking of sponges and its impact on the gentamicin concentration [11]. Taking this into account, our application technique did not include saline pre‐soaking of the sponge.

The primary spectrum of aminoglycoside antibiotics is directed against gram‐negative pathogens, whereas the skin flora, often responsible for the prosthetic graft infections, is predominantly represented by gram‐positive cocci (e.g., *Staphylococcus aureus* , *Staphylococcus epidermidis* ) [12]. The rationale behind choosing gentamicin in our setting was, that although its spectrum is mainly directed toward gram‐negative species, it is also effective against several gram‐positive strains [4]. Additionally, the majority of clinical evidence for local antibiotic application in cardiac surgery was obtained with gentamicin [10]. Our results have also demonstrated that, despite clinical signs of infection, pathogen agent identification and consequently the application of antibiogram‐driven anti‐infective treatment was not possible in the majority of cases.

mAFP explantation involving complete removal of the vascular graft and reconstruction of the axillary artery represents an alternative surgical technique. However, reopening of the wound and exposure of the axillary artery complicated by severe adhesions might result in brachial plexus injury, which can significantly limit the patient's quality of life and may also affect handling of the dLVAD [3]. Brachial plexus injury, with either sensory, motor, or sensorimotor deficits, was observed in more than one‐third of PGI patients after complete surgical removal of the vascular graft. In addition, surgical preparation of the axillary artery may be associated with a high risk of intra‐ and postoperative bleeding, which itself may increase the risk of wound infection [3]. The overall incidence of PGIs in our patient cohort does not support routine explantation of vascular graft prosthesis in all patients. Therefore, this approach should be reserved for cases of confirmed infection or for patients after heart transplantation, due to the risk for infection after initiation of immunosuppressive therapy.

In our study, we observed a survival benefit in the GICS group compared to the control group. This finding might be explained by an overall improvement in survival over time, as GICS implantation was introduced in the later period [13]. All patients who underwent mAFP explantation and demonstrated in‐hospital survival were included in the study. Patients who underwent an mAFP explantation for palliation or declined dLVAD therapy were excluded from the analysis.

## 5 Conclusion

The presented study demonstrated that the routine application of gentamicin‐impregnated collagen sponge during explantation of surgically implanted mAFP did not reduce the risk of prosthetic graft infections. Routine complete graft removal at mAFP explantation should be avoided due to the high risk of brachial plexus injury.

## Author Contributions

**Gaik Nersesian:** conceptualization, methodology, visualization, data curation, writing – original draft, writing – review and editing. **Maximilian Rauch:** data curation. **Daniel Lewin:** data curation, writing – original draft, writing – review and editing. **Leonard Pitts:** writing – review and editing. **Julius Kaemmel:** writing – review and editing. **Anna Stegmann:** writing – review and editing. **Julia Stein:** methodology, writing – original draft, data curation, writing – review and editing. **Yuriy Hrytsyna:** writing – review and editing. **Felix Schoenrath:** supervision, writing – review and editing. **Volkmar Falk:** supervision, writing – review and editing. **Evgenij Potapov:** conceptualization, methodology, supervision, writing – original draft, writing – review and editing. **Pia Lanmueller:** conceptualization, methodology, supervision, writing – original draft, writing – review and editing.

## Funding

The authors have nothing to report.

## Conflicts of Interest

F.S. received institutional grants from Novartis, Abbott, and institutional fees (speaker honoraria) from Abbott, Bayer, Novartis and Abiomed outside of the submitted work. V.F. declares relevant financial activities outside the submitted work with the following commercial entities: Medtronic GmbH, Biotronik SE & Co., Abbott GmbH & Co. KG, Boston Scientific, Edwards Lifesciences, Berlin Heart, Novartis Pharma GmbH, JOTEC GmbH and Zurich Heart in relation to educational grants (including travel support), fees for lectures and speeches, fees for professional consultation and research and study funds. E.P. declares payment related to proctoring, consulting, speaker fees, and institutional grants from Abbott, Abiomed, Medtronic, Recovery Therapeutics, and Cor Medical outside of the submitted work. P.L. received study and educational grants and speaker fees from Abiomed and Abbott outside the submitted work.

## Data Availability

The data that support the findings of this study are available on request from the corresponding author. The data are not publicly available due to privacy or ethical restrictions.
